# Active Hyperspectral Sensor Based on MEMS Fabry-Pérot Interferometer

**DOI:** 10.3390/s19092192

**Published:** 2019-05-12

**Authors:** Teemu Kääriäinen, Priit Jaanson, Aigar Vaigu, Rami Mannila, Albert Manninen

**Affiliations:** VTT Technical Research Centre of Finland Ltd, Vuorimiehentie 3, 02150 Espoo, Finland; jpriit@gmail.com (P.J.); aigar.vaigu@vtt.fi (A.V.); rami.mannila@vtt.fi (R.M.); almannine@gmail.com (A.M.)

**Keywords:** hyperspectral, lidar, remote sensing, supercontinuum, FPI

## Abstract

An active hyperspectral sensor (AHS) was developed for target detection and classification applications. AHS measures light scattered from a target, illuminated by a broadband near-infrared supercontinuum (SC) light source. Spectral discrimination is based on a voltage-tunable MEMS Fabry-Pérot Interferometer (FPI). The broadband light is filtered by the FPI prior to transmitting, allowing for a high spectral-power density within the eye-safety limits. The approach also allows for a cost-efficient correction of the SC instability, employing a non-dispersive reference detector. A precision of 0.1% and long-term stability better than 0.5% were demonstrated in laboratory tests. The prototype was mounted on a car for field measurements. Several road types and objects were distinguished based on the spectral response of the sensor targeted in front of the car.

## 1. Introduction

Hyperspectral remote sensing refers to a remote spectral detection of light, reflected or scattered from a target. Each pixel of a hyperspectral imager can contain hundreds of spectral channels, as opposed to the traditional three-color RGB cameras. Hyperspectral imaging has been widely applied in applications such as medical imaging and diagnostics [1], food safety inspection [2] and agriculture studies [3]. Hyperspectral cameras are usually dependent on ambient lighting. This limits the accuracy of the spectral signal since any variation in the illumination spectrum translates into a misinterpretation of the target response. 

Active hyperspectral sensing refers to a method where the investigated target is artificially illuminated by a broadband light source. A supercontinuum (SC) light source is most commonly used, however optical parametric oscillators (OPO) [4], as well as frequency combs [5], have also been used. SC is generated via spectral broadening of a short high-power monochromatic laser pulse in an optically non-linear material, usually an optical fiber [6]. The resulting light can have an optical bandwidth spanning over multiple octaves [7] but can still be directed and focused over long distances [8,9,10,11] as a regular laser. The potential applications for the technology include mineral survey, the automotive industry and agriculture.

The simplest case of an active hyperspectral sensor (AHS) or imager is a passive hyperspectral imager combined with an external light source to provide more light at the measured wavelengths [12]. However, as with passive illumination, the spectral radiant intensity of the external light source can drift. To extract the accurate spectral response of the target, a calibration target should be used at the target scene. A more attractive way is to calibrate the response with a calibration target only once and actively measure the spectrum of the outgoing beam for reference. Any drifts in the received signal caused by external illumination can be compensated by normalizing the received signal with the reference signal. This enables much more accurate measurements of the target spectral response compared to passive illumination, without the need for frequent calibrations of the spectral response. The active referencing is important when using SC illumination. The SC output is highly dependent on any variation in the fiber geometry and laser pulse properties. This usually results in large pulse-to-pulse variations and a drift of the output optical power and spectrum. 

The active monitoring of the outgoing beam can be realized with fast detection where the outgoing and received pulse are recorded with a single element. This approach has high requirements for the detection speed and cannot be used with integrating detectors. Nevertheless, fast detection will allow for combining the ranging measurement with the spectral measurement [13,14,15,16]. An alternative approach is to use two spectrometers, one monitoring the transmitted spectrum and another for the received spectrum. This approach requires two separate components to disperse the light, which results in a higher cost and complexity of the instrument. Thirdly, if the SC is already spectrally filtered prior to transmission, separate single-color point detectors can be used to detect both transmitted and received light. This approach poses a practical problem of implementing spectral scanning methods, which do not result in wavelength dependent illumination patterns over long distances. This would be the case if prisms or diffraction gratings were used for dispersion.

To date, relatively few prototypes of hyperspectral sensing using SC sources have been published and no commercial instruments exists. More work remains in both instrumental design and application studies in order to find the full commercial potential of the technology. In this work, we present an AHS prototype, based on a voltage-tunable near-infrared MEMS Fabry-Pérot interferometer (FPI) [17]. The FPI allows for a compact, cost-efficient and fast on-axis wavelength selection between 1300 nm and 1650 nm. We used the FPI to select the illumination band of the in-house built SC source. This approach enables the use of low-cost point detectors for both outgoing and received light. It also allows for more light to be transmitted in eye-safety critical applications compared to illumination of the whole bandwidth simultaneously. To demonstrate the approach, a compact battery-powered hyperspectral sensor was built, characterized and tested in the field.

This article is organized as follows. In Section 2.1 we present the system design of the prototype. Section 2.2 and Section 2.3 present the SC generation and the FPI characterization, respectively. In Section 3.1 we present profiling of the outgoing beam after the FPI and transmission optics. Based on the results, we extrapolate the spectral illumination pattern over long distances. In Section 3.2 we show the results from Allan-Werle deviation analysis of the received pulse energy and demonstrate how long period, calibration-free operation can be achieved with the active referencing. In Section 3.3 we present the results from the field measurements. The prototype is mounted on a test car and we present measurement results provided by the sensor, probing the road condition ahead of the moving vehicle. Finally, in Section 4 we conclude the work. 

## 2. Materials and Methods

### 2.1. Instrument Design 

**Figure 1:**
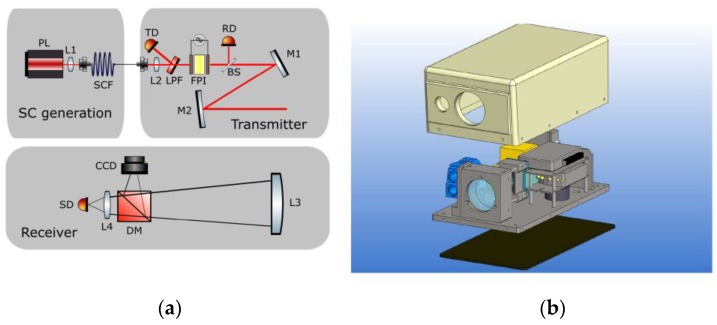
Schematic of the active hyperspectral sensor (AHS) instrument consisting of supercontinuum (SC), transmitter and receiver modules (**a**). PL = pump laser, SCF = supercontinuum fiber, L1 = short focal length lens for fiber coupling, L2 = short focal lens for focusing light through the Fabry-Pérot interferometer (FPI), LPF = long pass filter, TD = trigger detector, RD = reference detector, M1-M2 = convex and concave mirrors for beam expansion, L3 = light collecting lens, DM = dichroic mirror, SD = signal detector, L4 = focusing lens. CAD drawing of the prototype (**b**). The transmitter module is shown in blue.

The optical design of the AHS sensor is shown in Figure 1a. The SC module and FPI are explained in more detail in Section 2.2 and Section 2.3, respectively. The SC output is coupled to the transmitter module with a fiber connector. The wavelengths below 1300 nm are filtered out from the SC output with a reflective optical long-pass filter. The wavelength band <1300 nm, reflected from the long-pass filter, is measured by a fast InGaAs photodiode (Thorlabs FGA015, Newton, NJ, USA) connected to a comparator circuit to provide a timing signal for the data-acquisition. Wavelengths >1300 nm enter the FPI for spectral band selection. A small portion (1–5%) of the FPI-selected transmission band is reflected by a beam sampler onto the second InGaAs photodiode (Thorlabs FGA10) to provide a reference signal. The rest of the FPI-selected light is transmitted by the beam splitter. The transmitted light is expanded to approximately 3 mm 1/e^2^ diameter and collimated with convex (M1) and concave (M2) mirrors.

In optical systems with long light-propagation distances and wide wavelength bands, careful design is essential to avoid excessive chromatic aberration-related spectral dispersion of the light. Despite the drawback, a lens was used in the design to allow for axial beam propagation through the small aperture of the FPI. The effect on the beam propagation was characterized and is discussed in results. 

The collimated light from the transmitter module is overlapped with the field of view of the receiver module. The reflected light is collected by a spherical plano-convex lens (L3) with a 75 mm diameter. A dichroic mirror reflects visible wavelengths onto a CCD camera for visualizing the measured target. The received infrared light is focused onto the third InGaAs photodiode (Femto OE-300-IN-03-FST, Berlin, Germany). The detector has a programmable gain in order to account for varying target reflectance and distance. 

Both the signal and the reference detectors were limited in bandwidth to 1 MHz, even though the lengths of the actual SC pulses were less than 1.5 ns. The received pulses were stretched in the time domain in order to be digitized with slower and more cost-efficient analog-to-digital converters (ADCs). The signal was digitized with an open software FPGA-based development board (Red Pitaya STEM lab 125-14) with two 14-bit 125 MS/s ADCs. A custom C-program was used to remove the offset from the recorded waveforms and to integrate the pulse signals. Only the final integrated values were transmitted through Ethernet connection to a laptop for spectral analysis. A second multifunction data-acquisition card (NI myRIO) was used for general control of the instrument (laser, CCD, detector gain), as well as creating the FPI voltage ramp for the spectral scanning. The FPI voltage signal was synchronized with the acquisition using the TTL trigger from the trigger photodiode. A buffer was used in the FPGA to account for the delay between reading the trigger and the actual pulse. Even though the external laptop was used in this development work, both of the data-acquisition cards are capable of real-time standalone operation.

The mechanical design of the instrument is shown in Figure 1b. The instrument was enclosed in a 40 cm × 30 cm × 20 cm (l × w × h) water-proof seamless 3D-printed plastic housing. The total instrument weight was 9 kg. An easily replaceable tilted front glass was used to shield the transceiver optics. The metallic base plate can be used with adhesive heating pads for operation in cold conditions. The power consumed by the AHS sensor during the normal operation was 5 A with a 12 V power source.

### 2.2. Supercontinuum Generation

**Figure 2:**
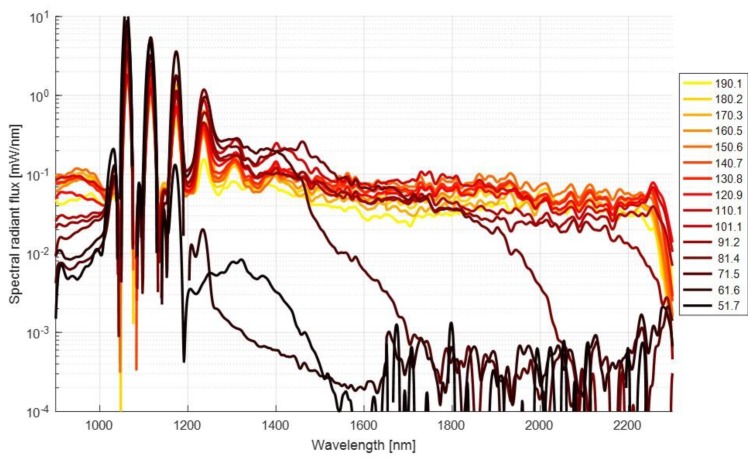
Spectral power density of the generated SC. The generation of longer wavelengths is more efficient with longer fibers, however, excessively long fiber results in a power-loss due to intrinsic fiber absorption. Short fiber lengths result in an inefficient spectral broadening.

The SC was generated with a 1064 nm pulsed microchip laser (Horus Laser HLX-I-F020-000, Limoges, France). The laser emits 25 kW peak power, 1.2 ns pulses at a 25 kHz repetition rate. The output from the laser is coupled to a low-cost multimode graded-index telecom fiber with a 50 μm core diameter and a 1310 nm zero dispersion wavelength (Corning InfiniCor50, New York, NY, USA). The spectral power density of the resulting SC spectra, generated in different fiber lengths, are shown in Figure 2. The resulting SC depends on the properties of the optical pump pulse as well as the fiber. Variation in coupling efficiency and any bending or movement of the SC fiber have a big impact on the resulting spectral power distribution. Moreover, the passive Q-switching of the pump laser results in a high fluctuation of the pump laser pulse at 1064 nm. The SC generation was tested with multiple fiber lengths to find the optimal spectral power distribution in the measurement bandwidth between 1300 nm and 1700 nm. The spectral measurements were conducted with a grating spectrometer (OceanOptics NIRQuest 512-2.5, Seminole, FL, USA). Based on the measurements shown in Figure 2, a fiber length of 100 m was used in the instrument. The SC generation is more efficient with longer fibers due to an increased probability for the nonlinear effects. However, if the fiber is too long, fiber absorption losses after 2250 nm start to decrease the output power. Too-short fiber results in an inefficient energy conversion to longer wavelengths. The compromise was made at 100 m, where the average radiant power within the desired measurement bandwidth of 1300–1650 nm was approximately 40 mW of the 500 mW pump power. 

### 2.3. Fabry-PérotInterferometer

**Figure 3:**
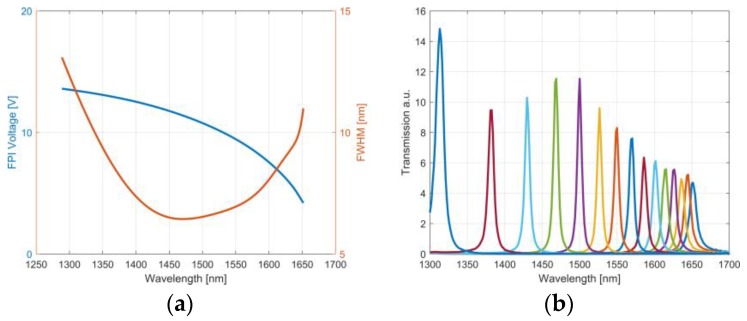
Tuning characteristics of the FPI. (**a**) The central wavelength and full-width at half maximum at a given voltage. The red curve shows the relationship between FPI voltage and FWHM. The blue curve shows the relationship between FPI voltage and the central bandpass wavelength. (**b**) Spectral transmission measured with a constant voltage interval, indicated by different colors.

The FPI consist of two opposing reflecting surfaces separated by an air gap, acting as a Fabry-Pérot resonator [18]. Due to the high reflectivity of the mirrors, only the light constructively interfering between the mirrors is transmitted. Thus, the gap between the mirrors determines the optical band. The miniaturized FPI, developed by VTT, can be tailored for a wide range of wavelengths and resolutions [17,19,20,21]. The gap and thus the wavelength band, is adjusted by applying voltage between two electrodes. The FPI used in this work had an aperture of 1.5 mm and operated with voltages between 0–13 V, resulting in a central wavelength shift between 1300 nm and 1650 nm. The tuning characteristics of the FPI are shown in Figure 3. The FPI wavelength and full-width at half maximum (FWHM) were determined using a grating spectrometer (OceanOptics NIRQuest 512, Seminole, FL, USA). The transmission data was fitted with a theoretical transmittance model of the Fabry-Pérot cavity to extract the central wavelength and FWHM. An automated routine characterized the FPI at 100 voltage values for an accurate description of the response. The non-linear voltage-central wavelength relationship of the FPI was fitted with a second-order polynomial function. The fit was used to generate a custom-form voltage ramp resulting in a linear wavelength scan. 

## 3. Results

### 3.1. Supercontinuum Propagation

In our previous work [8], the challenges of using refracting optics with the supercontinuum illumination at longer distances (100 m and over) were recognised. In this work, a refracting lens was needed after the supercontinuum to fit the beam through a small aperture of the FPI. In order to estimate the spectral uniformity of the illumination at different target distances, the beam was profiled with a thermal camera (NIT Tachyon 6400, Madrid, Spain) after the transmitter module. The beams spatial profile was measured at multiple distances between 0–12 m, scanning the central wavelength at each distance with 50 nm increments. The measured spatial beam profile was fitted with a two-dimensional Gaussian function. The beam profile resembled TEM00 spatial mode at all wavelengths with no signs of higher-order spatial modes. The resulting maximum energy density of the outgoing pulse was determined to be less than 0.8 μJ/cm^2^ at the wavelength of 1300 nm [22], which is well below the eye-safety limits dictated by the Finnish Safety and Chemicals Agency (TUKES).

**Figure 4:**
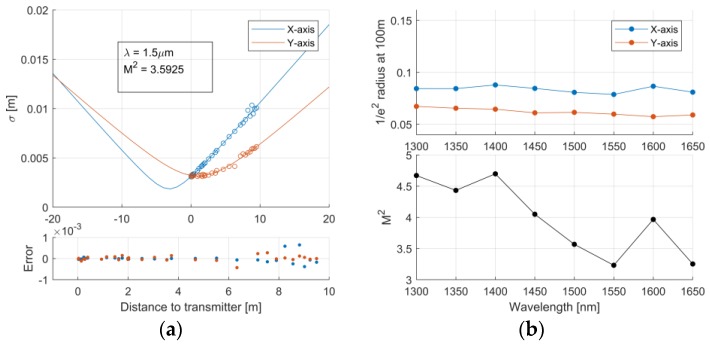
(**a**) Measured widths for single (1.5 μm) wavelength and a Gaussian fit Extrapolated 1/e^2^ diameter at 100 m and (**b**) the fitted M^2^ in function of wavelength.

The propagation of the beam was estimated by a Gaussian function. The results of the beam profiling with a fit for Gaussian propagation are shown for a single wavelength in Figure 4a. The ellipticity of the beam is the result of the astigmatism in the beam expander based on spherical mirrors. The average M^2^ value for both axes and all wavelengths was 4.0. The M^2^ value for the 8 wavelengths are shown in Figure 4b with the extrapolated 1/e^2^ diameter after 100 m of propagation. As can be seen, the fitted M^2^ decreases at higher wavelengths. This behavior is likely due to the fact that the SC generation is highly intensity-dependent. The power density threshold of the SC generation at shorter wavelengths is reached over a broader spatial area of the fibre, whereas the cascaded threshold for generating longer wavelengths is only reached at the center of the fibre. These lower M^2^ values compensate well for the larger divergences experienced by the longer wavelengths due to diffraction.

### 3.2. Receiver Response

**Figure 5:**
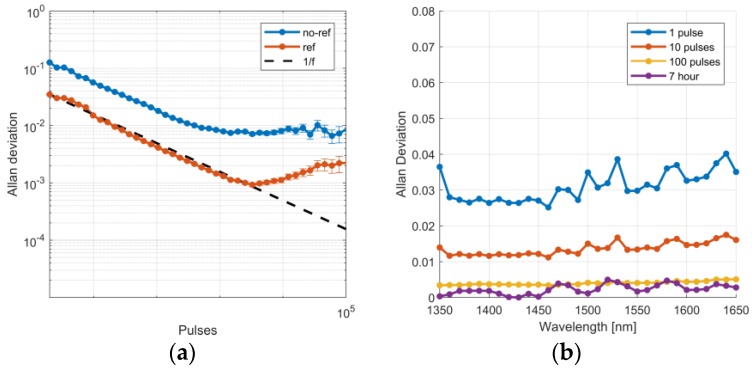
(**a**) Allan-Werle deviation of the measured pulse intensity with and without the reference signal. The center wavelength of the channel shown is 1500 ± 2.5 nm. Spectralon was used as the target at 5 m distance. The data was collected in 14 h. (**b**) The Allan-Werle deviation for averaged 1, 10 and 100 pulses as well as 7-hour values are shown for center wavelengths between 1350 and 1650 nm with 10 nm increments.

The Allan-Werle deviation analysis [23] was used to study the stability and noise performance of the spectral measurement. A white calibration target (Labsphere, Spectralon SRS-99-050, North Sutton, NH, USA) was placed 5 m from the instrument. The custom-form voltage ramp was applied to FPI at a 30 Hz repetition rate. The measurement was conducted continuously for 14 h in a temperature-controlled laboratory space. An Allan-Werle deviation plot of a single channel (1500 nm ± 2.5 nm center wavelength) extracted from the spectra is shown in Figure 5a. For comparison, both the raw intensity and the intensity corrected by the reference detector signal are shown. The noise performance and stability of the measurement are considerably improved by the active reference. Pulse-to-pulse deviation is reduced from 9% to 2% and the 7-hour Allan-Werle deviation is below 0.5% for all wavelengths. The analysis was repeated for the whole wavelength range with 10 nm increments in the central wavelength. The results are shown in Figure 5b. The 1, 10 and 100 pulses, as well as the 7-h Allan-Werle deviation are comparable with all wavelengths. The active reference efficiently replaces the otherwise frequent need for calibrations necessary to account for the SC drifts. The best possible precision of 0.1% is achieved with a 2000 pulse average. 

**Figure 6:**
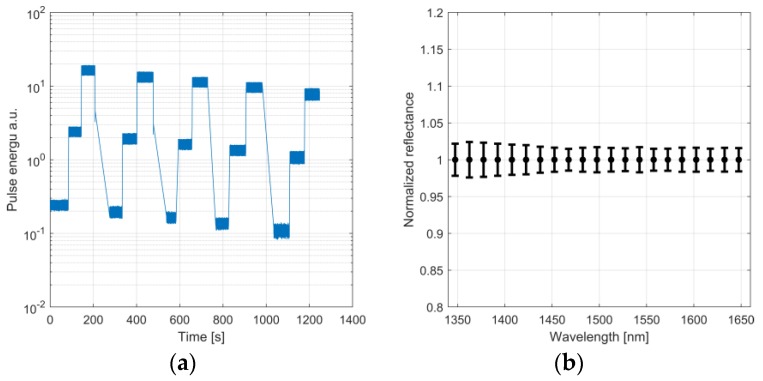
(**a**) Total intensity measured with three different gain levels and 5 levels of signal obstruction. (**b**) 1σ Standard deviation of intensity-normalized spectra.

Using the programmable gain of the detector, a total dynamic range of ~10^4^ can be achieved for the AHS sensor. The system response was measured by varying the signal level, both by obstructing the field of view and by changing the programmable gain of the detector. A total of 600,000 pulses were collected. The measurement conditions in these measurements were the same as in the Allan-Werle deviation analysis. Three different gain levels were used consecutively for five levels of physical FOV obstruction. The total received power levels are plotted in Figure 6a. With an ideal system, the intensity-normalized spectra should all give the same result. The average spectrum of all the measurements was used as a reference and the spectra recorded at different conditions were compared to this reference. The 1σ standard deviation of the spectra is shown in Figure 6b. The 1σ standard deviation was less than ±2.5% for all wavelengths. 

In addition to the instrumental drifts, the atmospheric absorption of the signal differs at varying conditions and distances, mainly due to water absorption. One solution around this is to limit the measurement to water absorption-free spectral windows. In this work however, the water absorption band was included in the spectral band since many interesting targets for the automotive industry, such as road friction, take advantage of these wavelenghts. For long-range spectral measurement with these wavelengths, compensation for the atmospheric transmission is necessary. This is however outside the scope of this work. 

### 3.3. Field Trials

**Figure 7:**
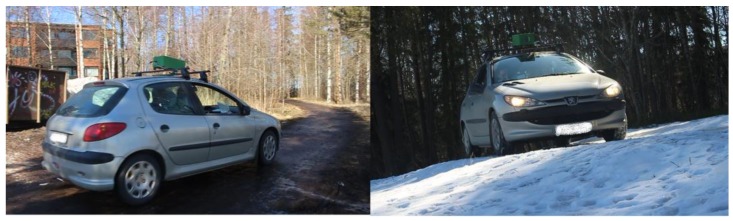
The sensor mounted on the test vehicle.

To test the robustness of the sensor in field environment, the instrument was mounted on a roof rack on top of a test car, as shown in Figure 7. The illuminated detection spot was directed at 10 m in front of the vehicle. The short distance was used in this pilot study to optimize the signal-to-noise ratio. The instrument was powered by a separate 12 V car battery. The test run included an off-road test track as well as urban road. With a lack of a calibration target, comparisons were done with similar road surfaces, mainly asphalt, at different time points to confirm that no significant drifts occurred. 

**Figure 8:**
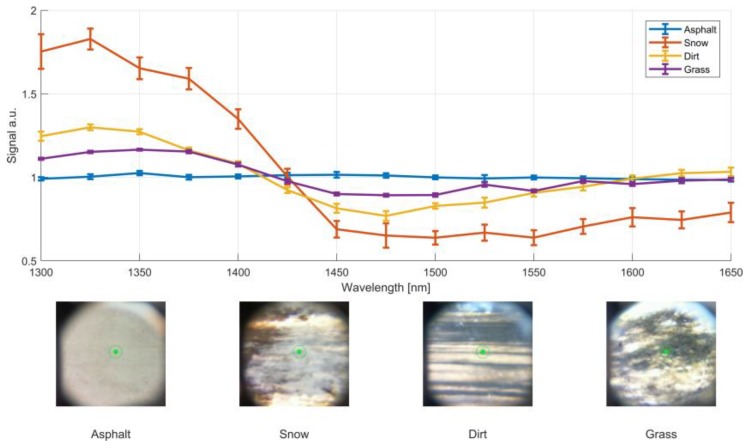
Selected spectra of road surfaces from the field-trial. Each spectrum is an average over 10 spectral measurements with a 100 Hz measurement rate. The standard error of the mean of the 10 spectra are shown as error bars. The test vehicle was driven with a speed ranging between 10–40 km/h.

**Figure 9:**
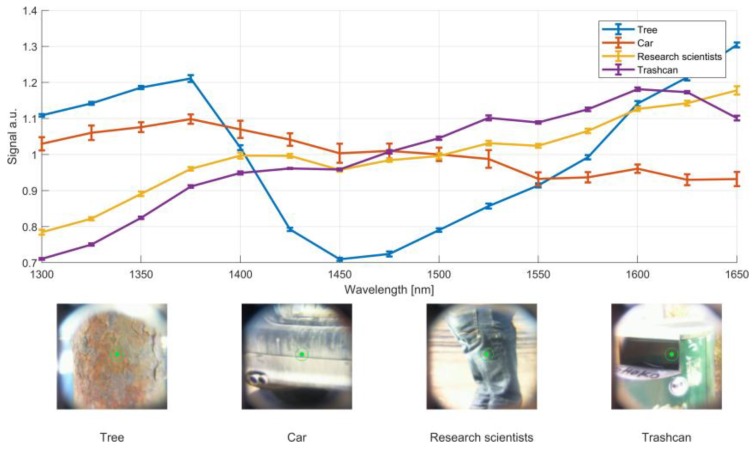
Selected spectra of objects from the field-trial. Each spectrum is an average over 10 spectral measurements with a 100 Hz measurement rate. The standard error of the mean of the 10 spectra are shown as error bars. The test vehicle was driven with a speed ranging between 0–10 km/h.

The prototype operated flawlessly during the whole test event. Some chosen spectra of different road types with the corresponding figure caption with the integrated CCD are shown in Figure 8. The spectra are an average of 10 individual spectra measured at a 100 Hz rate during the drive with speeds between 10–40 km/h. Each spectrum is normalized to a mean value of 1. The spectral response of the asphalt acted as the calibration surface and thus, is centered around one. In addition to road condition, other targets such as cars, pedestrians and obstacles produced a distinct spectral fingerprint. Selected spectra of objects are shown in Figure 9. The speed of the vehicle was reduced for object measurements, to only a few km/h. The shown spectra of a trash-can and a pedestrian were measured with the car being stationary.

## 4. Discussions

An AHS sensor based on MEMS FPI was built and demonstrated in both laboratory and in field. The FPI enables simple on-axis spectral filtering of the SC. For eye-safety limited applications, the FPI-based sensor allows for greater transmitted spectral power density due to the spectral scanning of the transmitted light compared to transmitting the whole SC. Furthermore, a simple active reference measurement using a single-point detection was shown to reduce the long- and short-term drifts considerably. 

The SC source was characterized for precise directionality. The large observed M^2^ values of the SC could be reduced by using single-mode fiber for SC generation. However, it seemed that the lower M^2^ of the longer wavelengths helped to uniform the illumination, correcting the difference in divergence of different wavelengths due to diffraction. Also, the use of multimode fiber has practical advantages on coupling the high-power pump light in to the fiber. The high peak-power pump pulses can damage the fiber end if the light is focused to the cladding instead of the fiber core. The larger core of the multimode fiber allows for more relaxed alignment. 

The small low-cost MEMS FPI optical bandpass filter was used for wavelength selection. The FPI filter does not refract different wavelengths and thus allows for light filtering before transmission of the SC. The FPI did not show any signs of degradation or change in tuning properties due to high-intensity laser light during this work. One of the limitations of using the FPI is the reduced bandwidth. The FPI used in this work covered merely 25% of the whole SC spectrum. Future development of the FPI filter might alleviate this limitation. Another practical limitation is the small aperture of the FPI that can govern the transmitters optical design. Finally, with FPI used only at the transmission in the presented prototype, the main detector is exposed to background radiation from the whole 350 nm FPI bandwidth. For this reason, it might be advantageous to synchronize a second FPI to filter ambient light. 

A best precision of 0.1% and long-term stability of 0.5% per spectral channel was demonstrated by using the Allan-Werle analysis. This performance, however, is limited to stable atmospheric conditions and received signal-strength. While this is not an issue in indoor applications, correction of the atmospheric attenuation is critical in outdoor measurements due to changes in relative humidity. Fog and dust particles also induce wavelength dependent scattering, affecting the spectral measurement.

With different detector gain levels and signal strengths, the overall 1σ standard deviation increased to 2.5% for all wavelengths, a factor of 5 greater than with the stable signal. It is possible, that small differences in the spectral uniformity affected the spectra measured with the light obstruction. More work is required in order to asses the reasons behind the non-ideal behaviour. 

The robustness of the instrument was put to test by mounting on a test vehicle. The sensor acquired spectra of the road 10 m in front of the vehicle. Several road conditions and objects were visually distinguished from the spectra. Future work should include building and integrating a model for automated recognition of the road types and common objects. In the case of autonomous driving or smart cars, the long-distance performance needs to be optimized. The sensor used in this work has been tested for only relatively short distances. With the current prototype, stationary solid targets, with reflectance larger than 0.1, have been measured with good SNR up to 50 m in lab conditions. To increase the distance, avalanche photodiodes could be employed in the design.

With the addition of a scanner, the AHS could be fused as a part of a sensory platform in self driving vehicles, providing target classification for objects, which are not recognized by conventional methods, such as lidar or camera. Future work will also investigate the potential applications for the sensor in other areas, such as mineral survey. 

## References

[1] Lu G., Fei B. (2014). Medical hyperspectral imaging: A review. J. Biomed. Opt..

[2] Feng Y.Z., Sun D.W. (2012). Application of hyperspectral imaging in food safety inspection and control: A review. Crit. Rev. Food. Sci..

[3] Adão T., Hruška J., Pádua L., Bessa J., Peres E., Morais R., Sousa J. (2017). Hyperspectral imaging: A review on UAV-based sensors, data processing and applications for agriculture and forestry. Remote Sens..

[4] Howle C.R., Stothard D.J., Rae C.F., Ross M., Truscott B.S., Dyer C.D., Dunn M.H., Fountain A.W., Patrick J., Gardner P.J. (2008). Active hyperspectral imaging system for the detection of liquids. Proceedings of the SPIE Defense and Security Symposium.

[5] Boudreau S., Levasseur S., Perilla C., Roy S., Genest J. (2013). Chemical detection with hyperspectral lidar using dual frequency combs. Opt. Express.

[6] Dudley J.M. (2010). Supercontinuum Generation in Optical Fibers.

[7] Silva F., Austin D.R., Thai A., Baudisch M., Hemmer M., Faccio D., Couairon A., Biegert J. (2012). Multi-octave supercontinuum generation from mid-infrared filamentation in a bulk crystal. Nat. Commun..

[8] Manninen A., Kääriäinen T., Parviainen T., Buchter S., Heiliö M., Laurila T. (2014). Long distance active hyperspectral sensing using high-power near-infrared supercontinuum light source. Opt. Express.

[9] Alexander V.V., Shi Z., Islam M.N., Ke K., Kalinchenko G., Freeman M.J., Ifarraguerri A., Meola J., Absi A., Leonard J. (2013). Field trial of active remote sensing using a high-power short-wave infrared supercontinuum laser. Appl. Opt..

[10] Islam M.N., Freeman M.J., Peterson L.M., Ke K., Ifarraguerri A., Bailey C., Wager M., Anthony A., Leonard J., Baker H. (2016). Field tests for round-trip imaging at a 1.4 km distance with change detection and ranging using a short-wave infrared super-continuum laser. Appl. Opt..

[11] Orchard D.A., Turner A.J., Michaille L., Ridley K.R., David H., Titterton D.H., Mark A., Richardson M.A. (2008). White light lasers for remote sensing. Proceedings of the SPIE Security + Defense.

[12] Nischan M., Joseph R., Libby J. (2003). Active spectral imaging. Linc. Lab..

[13] Hakala T., Suomalainen J., Kaasalainen S., Chen Y. (2012). Full waveform hyperspectral LiDAR for terrestrial laser scanning. Opt. Express.

[14] Li W., Sun G., Niu Z., Gao S., Qiao H. (2014). Estimation of leaf biochemical content using a novel hyperspectral full-waveform LiDAR system. Remote Sens. Lett..

[15] Powers M.A., Davis C.C. (2012). Spectral LADAR: Active range-resolved three-dimensional imaging spectroscopy. Appl. Opt..

[16] Du L., Gong W., Shi S., Yang J., Sun J., Zhu B., Song S. (2016). Estimation of rice leaf nitrogen contents based on hyperspectral LIDAR. Int. J. Appl. Earth Obs..

[17] Rissanen A., Guo B., Saari H., Näsilä A., Mannila R., Akujärvi A., Ojanen H., Piyawattanametha W., Park Y. (2017). VTT’s Fabry-Perot interferometer technologies for hyperspectral imaging and mobile sensing applications. Proceedings of the SPIE OPTO.

[18] Perot A., Fabry C. (1899). On the application of interference phenomena to the solution of various problems of spectroscopy and metrology. Astrophys. J..

[19] Akujärvi A., Guo B., Mannila R., Rissanen A., Piyawattanametha W., Park Y. (2016). MOEMS FPI sensors for NIR-MIR microspectrometer applications. Proceedings of the SPIE OPTO.

[20] Rissanen A., Akujärvi A., Antila J.E., Blomberg M., Saari H.K. (2012). MOEMS miniature spectrometers using tuneable Fabry-Perot interferometers. J. Micro Nanolithogr. MEMS MOEMS.

[21] Rissanen A., Mannila R., Tuohiniemi M., Akujärvi A., Antila J., Piyawattanametha W., Park Y. (2014). Tunable MOEMS Fabry-Perot interferometer for miniaturized spectral sensing in near-infrared. Proceedings of the SPIE MOEMS-MEMS.

[22] Jaanson P., Vaigu A., Kääriäinen T., Mannila R., Lehtomäki V., Manninen A., Singh U.N., Tzeremes G.D. (2018). A continuously tunable NIR laser and its applications in material classification. Proceedings of the SPIE Remote Sensing.

[23] Werle P.O., Mücke R., Slemr F. (1993). The limits of signal averaging in atmospheric trace-gas monitoring by tunable diode-laser absorption spectroscopy (TDLAS). Appl. Phys..

